# 

**Published:** 1878-02-20

**Authors:** 


					﻿a
af
a
a
Q
Q
*
a
a
O
><
o
a
a
a
°
; CO I
1 *—I ;
i a
Lu
o
i GO
s
t—4
<1
a
o
a
a
H
H
a
X
a
a
2-4
a
co
a
a
a
VOL. VIII. FEBRUARY 20, 1878. No 2^
THE SOUTHERN
MEDICAL RECORD.
A Monthly Journal of Practical,
‘Terms; $2per annum in advance. Club lyates, 5 copies	Single copies 20c.
“QUIVQUID PR^ECIPIES ESTO BREVIS,”
I
co
a
o
a I
H |
O
O
a
*
-2
a
o
■
02 !
U>
!
: a
•■0
a
1 >>
i o
, H
»—•
£
i t—t
I u.
i w
I H
I
■	S
I GO
O
i r
1 •—<
i o
■	i—t
I a
a
I P
c ESTABLISHED IN 1870.
ATLANTA, GA.:
Sunny South Steam Publishing House.
Address R. C. WORD, M. D., Managing Editor, Atlanta, Ga.
				

